# Supplementary material for: Anticandidal Potential of Stem Bark Extract from Schima superba and the Identification of Its Major Anticandidal Compound
Source: Molecules. 2019 Apr 22;24(8):1587. doi: 10.3390/molecules24081587 (PMC6515076; doi:10.3390/molecules24081587)
Supplement: Supplementary file 1 [file molecules-24-01587-s001.pdf]

molecules

MDPI

Supplementary Materials

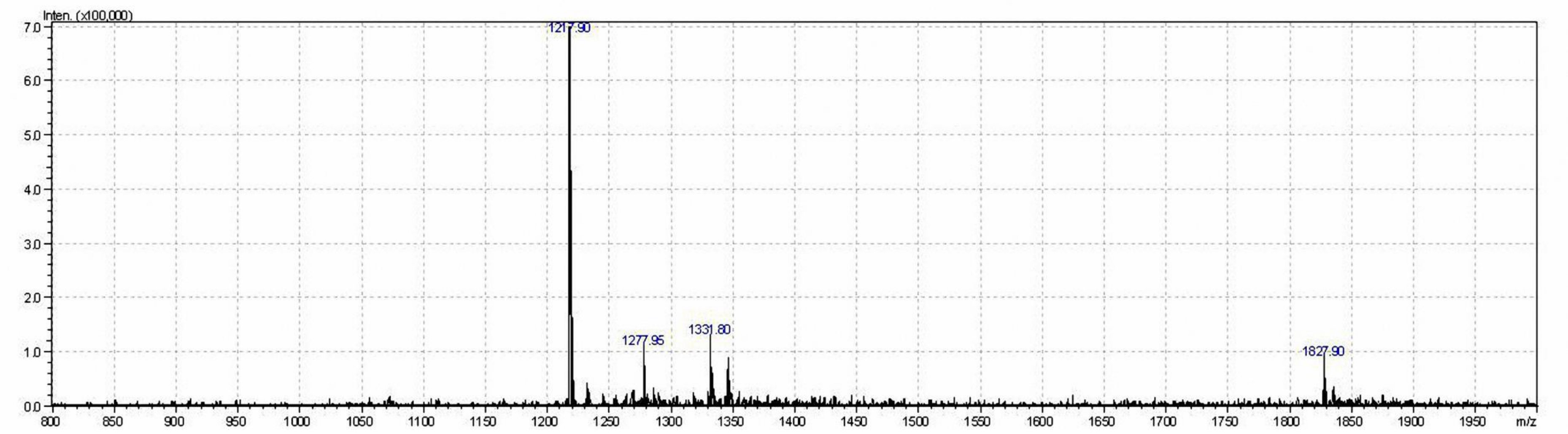

Figure S1. ESI-MS spectrum of compound **1.**

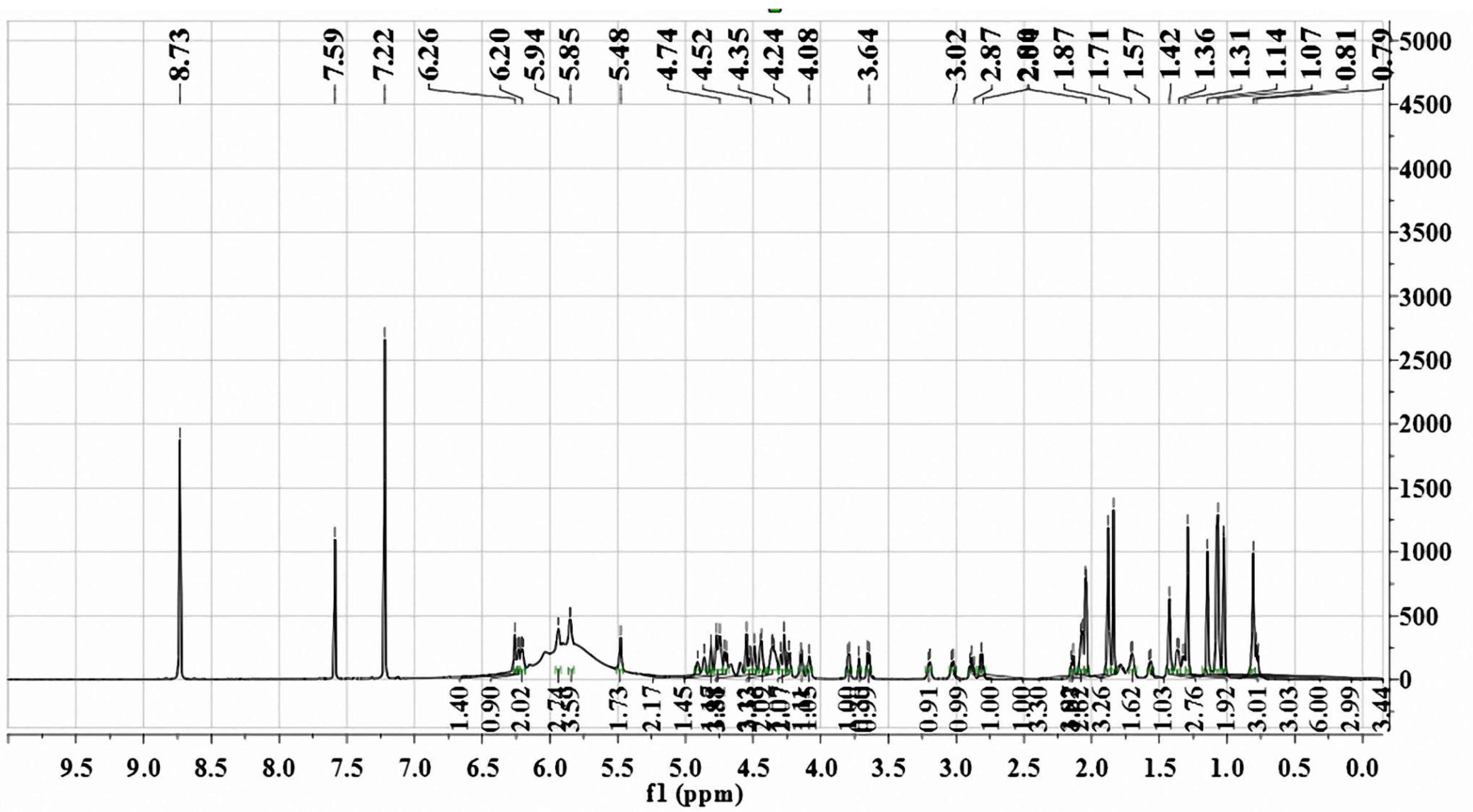

Figure S2. ^{1}H NMR spectrum of compound **1.**

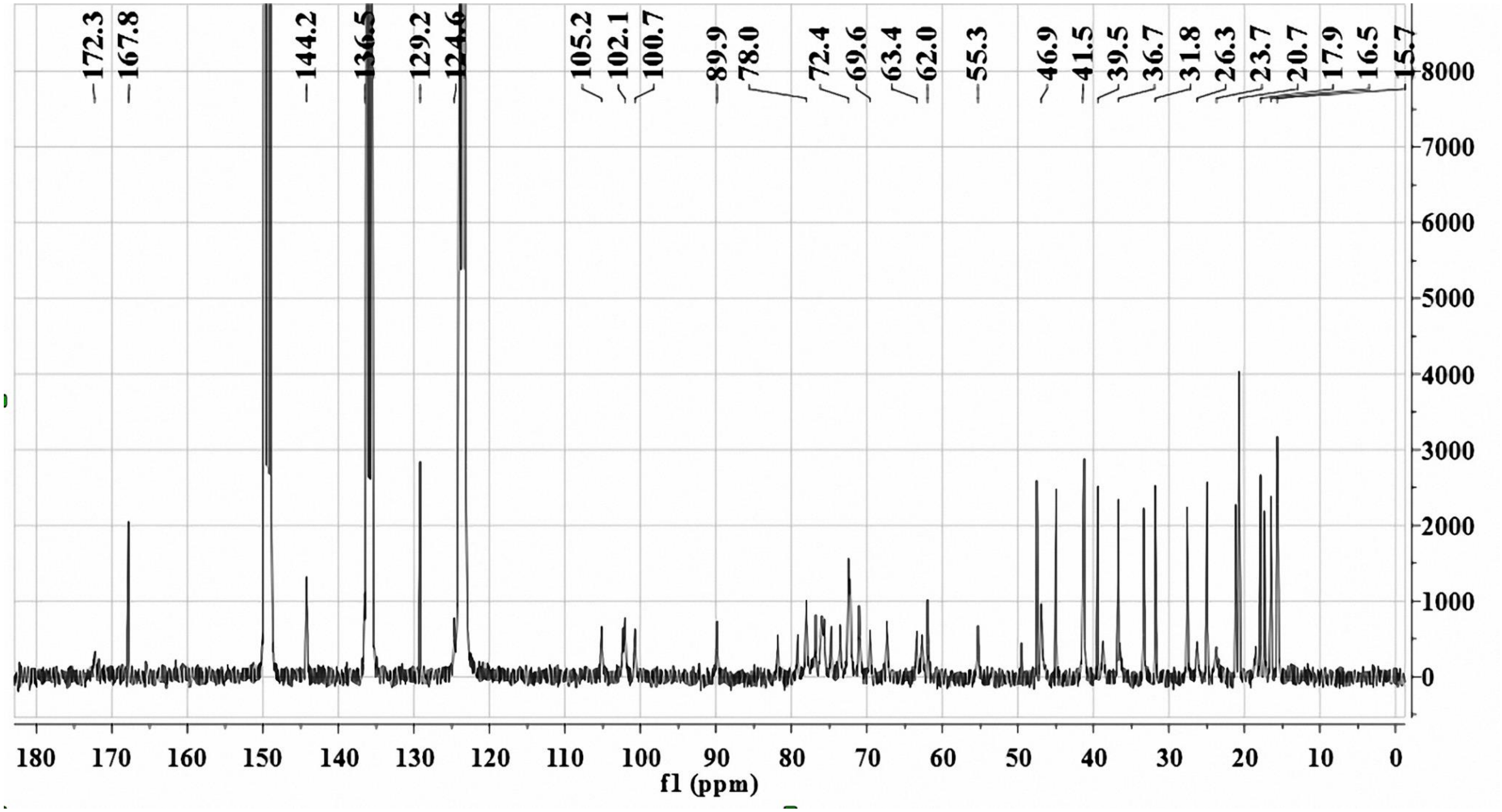

Figure S3. ^{13}C NMR spectrum of compound **1.**

Table S1. The 15 most down-regulated genes in a dose-dependent manner responding to SSE treatment.

ID	ID in GenBank	Annotation	Size (aa)	log_2 ratio	
				0.2 mg/mL	0.4 mg/mL
30515216	XP_019330880.1	Hypothetical protein (KEGG Orthology: ribosome biogenesis protein NSA2)	165	-3.12	-12.48
3638300	XP_720050.1	Hypothetical protein (KEGG Orthology: sulfonate dioxygenase)	423	-9.82	-10.61
3645419	XP_712951.1	Heat shock protein (HSP30p)	342	-8.0	-9.07
3642321	XP_716037.2	Hypothetical protein (KEGG Orthology: dimethylaniline monooxygenase)	500	-6.78	-7.96
3642587	XP_715780.1	Aquaporin water channel (AQY1p)	273	-6.44	-7.93
3635160	XP_723197.1	Thiamine thiazole synthase	354	-5.30	-7.91
3641921	XP_716458.1	4-amino-5-hydroxymethyl-2-methylpyrimidine phosphate synthase	339	-5.50	-7.81
3641757	XP_716605.1	Hypothetical protein (KEGG Orthology: dimethylaniline monooxygenase)	536	-6.50	-7.69
3636132	XP_722228.1	Sulfate adenylyltransferase	527	-5.0	-7.26
3643318	XP_715020.1	High-affinity iron permease	381	-6.46	-7.11
3634945	XP_723459.2	Alternative oxidase (AOX2p)	365	-3.62	-7.09
3642078	XP_716238.1	Hypothetical protein (KEGG Orthology: sulfonate dioxygenase)	385	-5.45	-6.67
3645007	XP_713317.1	GPI-linked cell wall protein (RBT5p)	241	-5.40	-6.60
3645348	XP_713038.2	Inorganic phosphate transporter	545	-1.69	-6.50
3645347	XP_713037.1	Methyltransferase domain (BTA1p)	752	-2.63	-6.46

Table S2. The 15 most up-regulated genes in a dose-dependent manner responding to SSE treatment.

ID	ID in GenBank	Annotation	Size (aa)	log_2 ratio	
				0.2 mg/mL	0.4 mg/mL
3636547	XP_721815.1	Membrane-associating domain (MRV4p)	256	5.92	6.63
3636546	XP_721814.1	Hypothetical protein (MRV3p)	195	3.82	6.52
3638048	XP_720385.1	Sugar transporter (HGT12p)	526	1.65	5.64
3638886	XP_719507.1	Cu-containing superoxide dismutase (SOD5p)	228	5.33	5.55
30515234	XP_019330904.1	Metallothionein (CRD2p)	76	3.89	4.63
3643211	XP_715162.1	MFS transporter	376	3.48	4.30
3643209	XP_019331073.1	Sugar transporter (HXT5p)	562	2.53	4.28
30515097	XP_019330757.1	Hypothetical protein (Protein of unknown function)	87	3.73	4.03
3647880	XP_710528.1	Member of a family of telomere-proximal genes of unknown function (TLO11p)	169	3.23	3.97
3647320	XP_711076.1	Phenylpyruvate decarboxylase	629	3.69	3.97
3635607	XP_722755.1	Secretory protein (DAG7p)	251	3.27	3.81
3641618	XP_716740.1	NAPDH dehydrogenase (OYE23p)	406	1.83	3.73
3638359	XP_720039.1	Hypothetical protein (Protein of unknown function)	271	3.57	3.72
3638325	XP_719950.2	Glutathione-independent methylglyoxalase	236	2.20	3.71
3639861	XP_718476.1	SPX domain-containing inorganic phosphate transporter	1006	3.23	3.70

Table S3. comparison of gene expression including up-regulation and down-regulation by RNA-seq and Real-time PCR.

ID	Annotation	$\log_2$ ratio by RNA-seq		Fold change by real-time PCR	
		0.2 mg/mL	0.4 mg/mL	0.2 mg/mL	0.4 mg/mL
3638048	Sugar transporter (HGT12p)	1.65	5.64	3.72	3.8
3636838	MFS transporter (HOL1)	0.92	1.72	3.7	2.98
3646427	Aldo-keto reductase (CSH1)	1.18	1.88	7.06	8.44
3640751	Alcohol dehydrogenase	1.71	2.16	7.76	10.46
3639294	Heme oxygenase (HMX1p)	-0.93	-1.74	-1.49	-4.04
3644402	Heat shock protein (ASR1)	-1.42	-2.0	-4.58	-20.56
3635222	Trifunctional fatty acid synthase subunit	-1.12	-1.66	-4.0	-16.13
3640144	Omega-class glutathione transferase	-1.05	-2.12	-3.42	-26.26

Table S4. The primer sets of *C. albicans* genes were used in real-time RT-PCR for quantitation of gene expression.

Gene ID	Protein	Primer sequence (5’-3’)
18SrRNA	18S rRNA	Forward: CGATGGAAGTTTGAGGCAAT<br>Reverse: CACGACGGAGTTTCACAAGA
3638048	HGT12	Forward: TGTTGGTTCCTTGATTGCC<br>Reverse: GGCTCTACCTGCAATAAGCAAT
3636838	HOL1	Forward: CAGCAGCTACTTCAAATGACG<br>Reverse: CCCCATCCAATACCCAAA
3646427	CSH1	Forward: AAAGCATATCTTAGCCGCAG<br>Reverse: GCCAATCCTTGTTCAACAAC
3640751	ADH2	Forward: GAATACTGTCAATCAGGTGCTG<br>Reverse: GGATTCTGGCAGCTTGAAC
3639294	HMX1	Forward: AAAGGCCTTGTACAGACAGCT<br>Reverse: TCTTGCTCGGCTTTACCA
3644402	ASR1	Forward: CGGATCATCAAACACTGCTAGT<br>Reverse: CCAGATCCATAACTTCCACTTG
3635222	FAS2	Forward: CAAAGCAAGAAGAGAGTACCCC<br>Reverse: CCTTGACTGGTTCATCTGGAAT
3640144	ECM4	Forward: GGCATATGGATGACAAAGGG<br>Reverse: CGTATTCTGGTTCAGCCTTGA
